# Novel Two-Dimensional Mechano-Electric Generators and Sensors Based on Transition Metal Dichalcogenides

**DOI:** 10.1038/srep12854

**Published:** 2015-08-04

**Authors:** Sheng Yu, Kwesi Eshun, Hao Zhu, Qiliang Li

**Affiliations:** 1 Department of Electrical and Computer Engineering, George Mason University Fairfax, VA 22033 USA

## Abstract

Transition metal dichalcogenides (TMDCs), such as MoS_2_ and WSe_2_, provide two-dimensional atomic crystals with semiconductor band gap. In this work, we present a design of new mechano-electric generators and sensors based on transition metal dichalcogenide nanoribbon PN junctions and heterojunctions. The mechano-electric conversion was simulated by using a first-principle calculation. The output voltage of MoS_2_ nanoribbon PN junction increases with strain, reaching 0.036 V at 1% strain and 0.31 V at 8% strain, much larger than the reported results. Our study indicates that the length, width and layer number of TMDC nanoribbon PN junctions have an interesting but different impact on the voltage output. Also, the results indicate that doping position and concentration only cause a small fluctuation in the output voltage. These results have been compared with the mechano-electric conversion of TMDC heterojunctions. Such novel mechano-electric generators and sensors are very attractive for applications in future self-powered, wearable electronics and systems.

Mechanical energy is one of the most ubiquitous energy sources in the environments and is readily accessible from the human activities. Therefore, the conversion of mechanical energy into electricity offers an immediate, stand-alone power support for remote control systems, wearable electronics, wireless sensing and remote battery charging^1^,^2^,^3^,^4^,^5^,^6^. Piezoelectric transducer is the most distinguished technique for harvesting vibration based energy by its high power output and relatively low-cost manufacturing^7^. Recently, the piezoelectric properties of nanowires have been widely studied for potential applications in sensors, transducers, energy conversion and electronics^8^,^9^. The power generators based on piezoelectric nanostructures were successfully designed and fabricated^10^,^11^,^12^. The coupling of semiconductor and piezoelectric properties in one-dimensional (1D) ZnO nanowires (NWs) in a nanogenerator was reported with excellent power conversion efficiency from 17% to 30%^13^. However, the difficulty in aligning 1D ZnO NWs may hinder high-performance applications in Nanoelectromechanical Systems (NEMS)^13^,^14^,^15^,^16^.

Two-dimensional (2D) layered materials, such as hexagonal boron nitride (h-BN) and transition metal dichalcogenides (TMDCs)^16^,^17^,^18^, have gained considerable attentions for electronic applications. Their mechanical properties, possible application in body movement, muscle stretching and blood vessel contraction^19^,^20^, have also been studied. The MoS_2_ monolayer, a typical member of 2D TMDCs, becomes piezoelectric after exfoliation from the bulk crystal whereas the inversion symmetry is broken^21^,^22^. The asymmetry leads to valley polarization caused by valley-selective circular dichroism. This attracts potential applications in valleytronic devices^23^,^24^. Therefore, MoS_2_ nanostructures have become promising in NEMS application^25^,^26^ in nanopiezotronics, a rapidly emerging field.

The piezoelectric properties and applications of MoS_2_ monolayers, such as mechanical energy harvesting and piezotronic sensing, have very recently been explored experimentally^25^,^26^. Angular dependence of inversion symmetry has been measured by optical second-harmonic generation (SHG)^27^, indicating that it is mostly broken along armchair direction while preserves along zigzag direction. The absence of centrosymmetry endows MoS_2_ monolayer with piezoelectricity along the armchair direction^19^. An open-circuit voltage of 18 mV has been demonstrated at 0.53% strain along the armchair direction^26^ in a MoS_2_ monolayer of a dimension of 10 μm in length and 5 μm in width. This output voltage is quite small. Enhancement of output performance is very important for further application of 2D materials in mechanical-to-electric generators.

In this work, we report a novel mechano-electric conversion device based on TMDC nanoribbon PN junctions and heterojunctions. As shown in Fig. 1(a), a TMDC nanoribbon mechano-electric generator can be used to convert human muscle stretching power to support wearable electronics. Our first-principle calculation has shown that high output voltages, 0.036 V and 0.31 V at 1% and 8% strain, respectively, can be achieved in a 1.5 nm × 5 nm MoS_2_ nanoribbon PN junction. In consideration of the small size of nanoribbon, this mechano-electric generator has a high conversion voltage and its performance can be improved significantly by a series of connection^26^. Our study indicates the mechano-electric conversion of 2D TMDC PN junction is better than that of the heterojunction. This work is the first study of designing 2D TMDC junctions for application in high-performance mechano-electric conversion, suggesting a new way of using 2D TMDCs for future nanogenerators and sensors.

## Methods in Simulation

In this work, the energy diagrams of 2D TMDC nanoribbon PN junctions and heterojunctions have been calculated by first principle calculations carried out by the density functional theory (DFT) in Virtual Nanolab ATK package^28^. The n-type and p-type TMDCs are achieved by substitutional doping. The Localized Density Approximation (LDA) exchange correlation with a Double Zeta Polarized (DZP) basis was used with a mesh cut-off energy of 150 Ry^29^. We used 1 × 1 × 50 Monkhorst-Pack k-grid mesh in this simulation with more k-points in transport direction^30^. All atomic positions and lattice constants were optimized by using the Generalized Gradient Approximations (GGA)^31^ with the maximum Hellmann-Feynman forces of 0.05 eV/Å. Pulay-mixer algorithm was employed as iteration control parameter with a strict tolerance value of 10^−5^ 32. The maximum number of fully self-consistent field (SCF) iteration steps was set to 1000^29^. The electronic temperature was set to 300 K for all the simulations. The self-consistent field calculations were checked strictly to guarantee fully converging within the iteration steps.

In order to clearly illustrate the design and characteristics of TMDC junction mechano-electric converters, the results are reported as follows: (1) The intrinsic piezoelectricity of 2D infinite MoS_2_ monolayer was studied. (2) The PN junction-based device electric output performance was evaluated. (3) Effects of sizes (width, length and layer number) on output voltages were investigated. (4) The fluctuation in output voltage induced by various doping positions and concentrations was studied. (5) The mechano-electric conversion of TMDC nanoribbon heterojunctions were studied and compared with that of PN junctions. Finally, the mechano-electric conversion of various designs based on 2D TMDCs was analyzed and compared.

## Results and Discussion

**Figure 2:**
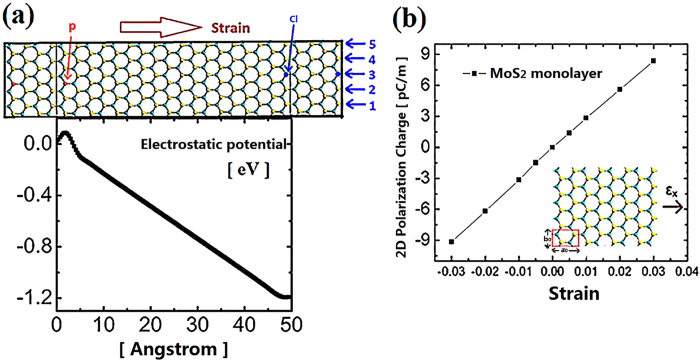
(**a**) Configuration of our designed device, where S atoms are yellow; Mo atoms are cyan; P atom is red and Cl atom is blue. The left and right rectangles represent the left and right electrode. P replaces S at the middle position of left edge of center region. Cl replaces S at the middle position of right edge of center region. (**b**) The electrostatic potential as a function of position in the central region under zero strain. (**c**)The polarization charge as the function of strain along in-plane armchair direction

The basic MoS_2_ monolayer PN junction was configured as Fig. 2(a). The model is divided into three regions: left electrode, right electrode, and central region. The central scattering region consists of 5 × 9 unit cells: the width is composed of 5 periodic unit cells in zigzag direction and 9 basic lattice lengths are included in armchair direction, which is designed as transport direction in this study. In the transport direction Dirichlet boundary condition was applied on the two opposite electrodes, in which the electric potential was held homogeneously across the boundary. Neumann condition was employed on the other two directions, in which the electric field was held homogeneously at the boundary. MoS_2_ nanoribbon exhibits intrinsic semiconducting property and strongest piezoelectricity along the armchair direction while metallicity and highly crystal inversion symmetry are demonstrated in zigzag direction^33^,^34^. The coupled semiconducting and piezoelectric properties are responsible for the mechanism of power generator^35^. Substituting sulfur (S) by chlorine (Cl) shifts the Fermi level towards conduction bands, resulting in n-type doping while the inverse p-type doping is realized by the replacement of phosphorus (P). The impurity density on both sides is chosen to be 10^13^ cm^−2^ within reasonable computational burden^36^. Fig. 2(b) displays the electrostatic potential as a function of position in the unstrained central region. As shown it is decreasing monotonically along the transport direction and the electrostatic potential dropping (EPD) is 1.174 eV at right edge with respect to the left counterpart. This is consistent with our design that p-type is realized at the left side while the right side is n-type.

## Intrinsic piezoelectricity of 2D MoS_2_ monolayer

Firstly, the intrinsic piezoelectricity of MoS_2_ monolayer is investigated. Noncentrosymmetric lattice structure is necessary for a material to be piezoelectric^37^,^38^. The three-dimensional (3D) bulk stacked-layer h-BN and 2H-TMDC crystals are centrosymmetric due to their experimentally observed antiparallel stacking sequence^39^. However, the two dimensional (2D) monolayer of TMDCs, such as MoS_2_, WSe_2_, WS_2_, MoSe_2_ etc., which have been successfully fabricated by exfoliation from their 3D bulk materials^40^,^41^,^42^,^43^, exhibits noncentrosymmetric crystal structure^22^. This noncentrosymmetry stems from the particular dislocated stacks of the different layers composed by chalcogen atoms and transition elements and accordingly results in the absence of inversion center. As a typical member of TMDCs, 2D MoS_2_ monolayer is naturally piezoelectric. Figure 2(c) shows its polarization charge as the function of strain applied along in-plane armchair direction. In this work, the strain is evaluated as the lattice changing percentage. We defined ε_x_ ≡ Δa_0_/a_0_, where Δa_0_ is the increase of lattice constant a_0_ due to the strain. a_0_ = 5.47 Å, signifying the lattice constant along the transport direction (armchair direction). The coefficient e11, defined as the slope of linear fit line for charge vs. strain curve, reveals the change in polarization along armchair direction per area by strain^19^. Our estimation of e11 is 2.98 × 10^−10^ C/m, which is very close to the experimentally reported 2.90 × 10^−10^ C/m^25^.

## PN junction MoS_2_ nanoribbon based device

**Figure 3:**
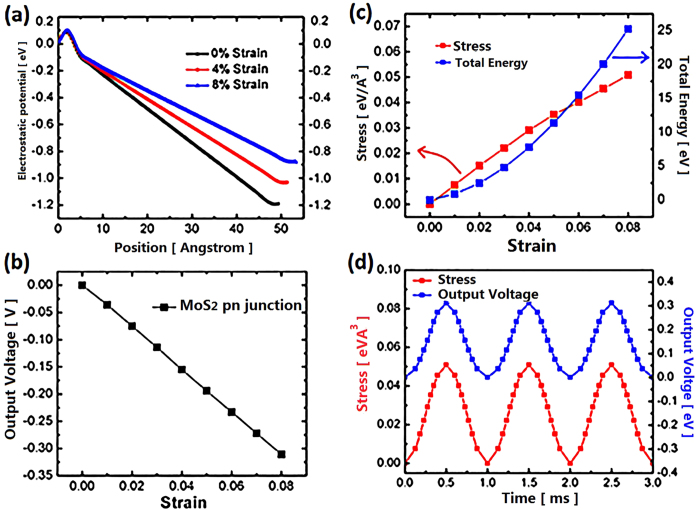
(**a**) 0%, 4% and 8% tensile strain modulated electrostatic potential along transport direction in the central region. (**b**) The output voltage as a function of strain. The negative value in our study denotes the electrical potential in left electrode is higher than right electrode. (**c**) The stress and total energy of the central region of our model as a function of strain. (**d**) Red: stress in sine waveform-time domain is laterally applied on our device. Blue: the output voltage response.

Secondly, the electronic property of our model under lateral strain has been simulated. The strain given by ε = (L–L_0_)L_0_ is initially applied along transport direction, where L_0_ and L is the equilibrium length along the transport direction of the unstrained and strained device, respectively. Fig. 3(a) reveals the electrostatic potential distribution along the transport direction in the central region for the device applied by 0%, 4% and 8% tensile strain, respectively. The central region was extended from 49.2 Å to 53.2 Å in length by 8% strain. As shown the structure under 8% strain has the smallest EPD. EPD reduces from 1.174 V for unstrained structure to 0.878 V for the structure under 8% strain. Fig. 3(b) demonstrates the output voltage as a function of strain applied along the transport direction. The absolute value of output voltage is linearly increasing by larger strain. The maximum output voltage is 0.310 V in the case of 8% strain. The negative value denotes that the electrical potential at left electrode is higher than that of right electrode and therefore the left side serves as the anode while the right counterpart is the cathode in our device. Our study suggests a nano-generator with excellent performance, which has output of ~20 mV in small size (5 nm × 1.5 nm) under 0.5% strain. This indicates significantly enhanced performance by doping and PN junction based device over undoped MoS2 nanosheet in large area^26^. This tremendous improvement in output is attributed to the strongly enhanced polarization between bipolar atoms induced by the coupled built-in electric field and external strain.

Next, we investigate the mechanical property of our device based on PN junction. Fig. 3(c) demonstrates the variation of total energy with uniaxial strain applied along transport direction. The total energy (E_total_) is increasing monotonically as the increasing strain (ε). The slope of this curved line given by 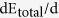
ε is also rising by the increasing strain. The evolution of the stress with strain is estimated by the mathematic expression: 
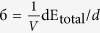
ε, where V is the volume of our sampled system^44^. The orthorhombic cube with the total volume of 9.6 nm^3^ was sampled in our study. The stress required for deformation intensity denoted by strain ε is increasing monotonically with the larger strain. The stress vs. strain relation keeps good linearity within the small strain range 0 ≤ ε ≤ 3% and the elastic modulus C is keeping constant by the expression: C = *d*б/*d*ε. Previous report indicated that this parameter can remain constant within small strain (−2% ≤ ε ≤ 2%) for MoS_2_ monolayer^45^,^46^. For the larger strain from 4% up to 8%, this relation slightly deviates from linearity and accordingly the elastic modulus C reduces. Fig. 3(d) shows the output voltage response for our device under laterally applied stress in Sine waveform-time domain. The periodic time of our dynamic stress is 1ms. Therefore a proper assumption can be suggested that there is negligible delay between input force and output voltage phase^47^. Experimentally, the stress can be realized by bending the substrate periodically^26^. For the mathematic expression of time dependent stress, we deduce it as following:
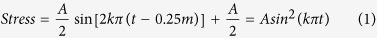
where A is the maximum stress with the value of 0.051 eV/ Å^3^, which induces 8% strain. As shown the maximum output voltage ~0.310 V is reached at the maximum stress.

## Effects of Sizes

**Figure 4:**
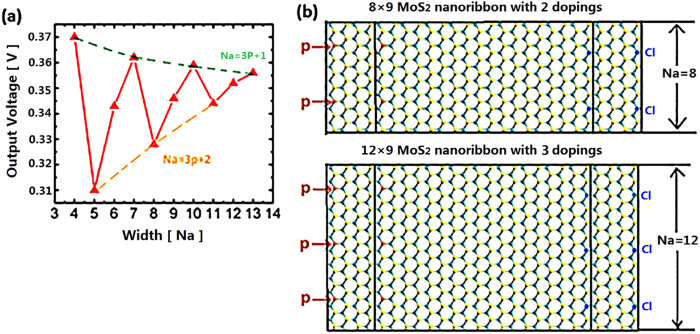
The investigation on width effect. (**a**) The evolution of output voltage with MoS2 nanoribbon width. All the structures are keeping one doping atom at each side. (**b**) Configuration of nanoribbon with width (Na = 8, Na = 12) by 2 doping and 3 doping atoms, respectively.

The evolution of the output voltage with the nanoribbon width (N_a_) was also investigated. The nanoribbon width is denoted by periodical number of unit cells in zigzag direction vertical to the transport direction. For each structure with incremental width, one doping atom was kept at the center of lateral edge. The length of nanoribbon was kept 9 periodic unit cells in the transport direction. As displayed in Fig. 4(a), the output voltage oscillations are observed for the narrow ribbons, and those nanoribbons of N_a_ = 3p + 1 (where p is an integer) have larger output than the neighboring two nanoribbons. With increasing width, the output voltage finally converges to a constant value ~0.355 V. The enlarged size will attenuate the doping concentration, and might lead to unexpected impact on the performance of our device. To clarify this issue, two pairs of phosphorus and chlorine doping atoms are introduced in 8-width structure (Fig. 4(b)), and also, three pairs of doping atoms are introduced in 12-width nanoribbon (Fig. 4(c)). The doping concentration of these structures are keeping the same as 4-width structure with one pair of doping atoms. The output voltage for these two structures are 0.328 V and 0.346 V, indicating slight difference with one doping structure of 8-width (0.328 V) and 12-width (0.352 V), respectively. It should be noted that the output of the structure with low doping concentration is slightly higher than that of the highly doping structure. This issue will be discussed in part **doping effect.**

**Figure 5:**
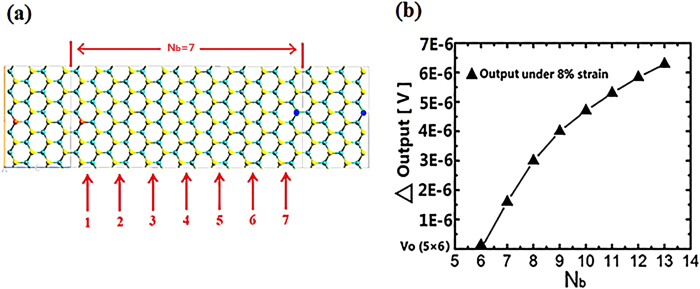
The investigation on length effect: (**a**) Definition of length for MoS_2_ nanoribbon. (**b**) Comparison of the output voltage for structures with different lengths. The output of 5 × 6 is 0.3103446 V, setting as the reference value.

In addition to the width effect, we also investigate the influence of nanoribbon length on the output performance. As displayed by Fig. 5(a) the length of nanoribbon is defined by the periodic lattice number (N_b_) of the central region in the transport direction (armchair direction). The width number (N_a_) is kept constant value of 5 for all the investigations on length effect. The output voltage for the device under 8% strain as a function of N_b_ is shown in Fig. 5(b). The output voltage of the nanoribbon with N_b_ = 6 is 0.3103446 V, setting as the reference value. The output voltage is slightly increasing by the order of magnitude of 10^−6^ V with increasing N_b_. The rising rate (∆Output/∆N_b_) reduces as larger N_b_, indicating that the output will saturate to a constant value under sufficiently large N_b_. Our study indicates that the structure length has negligible effect on the output voltage.

**Figure 6:**
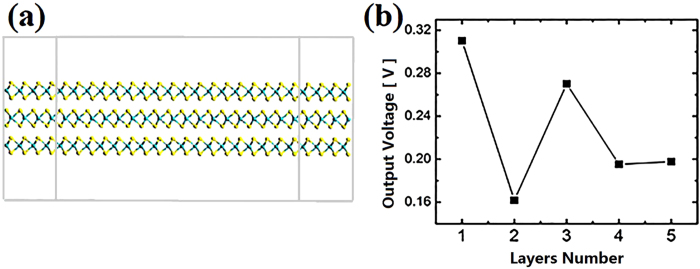
The investigation on layers effect. (**a**) Configuration of 3 layers MoS_2_ mechano-electric converter. Each layer is doping by a pair of P and Cl, respectively. (**b**) Output voltage for device under 8% strain as a function of layer number.

We also investigated the output voltage as a function of layer number of MoS_2_ stacked structure. Fig. 6(a) shows the configuration of 3 layers MoS_2_ mechano-electric converter. Each layer is doping by a pair of P and Cl, respectively. P replaces S at left side while Cl is doping at right side. As demonstrated by Fig. 6(b), the output voltage exhibits a fluctuant behavior as increasing layers. It reaches maximum of 0.310 V by single layer, while is reducing significantly to 0.16 V by 2 layers stacked structure. As limited by the simulation complexity and its converging difficulty, we only put forward our investigation to 5 layers. However, a reasonable speculation can be made that the output voltage finally converges to a constant value as increasing layer number, which is trended similarly as that tuned by increasing width. In experiments the output voltage of undoped MoS_2_ in large area shows a proportional relation with the second-harmonic generation (SHG) intensity for stacked structures: MoS_2_ flakes stacked by odd number of layers exhibited strong piezoelectricity along armchair direction, while the output voltage disappeared for even number of layers^26^. Our study shows that the structures with even number of layers still have strong output, indicating a distinct underlying physical principle with the device by undoped nanosheet. It should be noted that as the larger size (Larger width, length and layer number), the output voltage of our device tends to converge to a constant value. Our study suggests a mechano-electric generator with weak dependence on dimension and size, which is exceedingly favourable for industrial application.

## Doping effect

**Figure 7:**
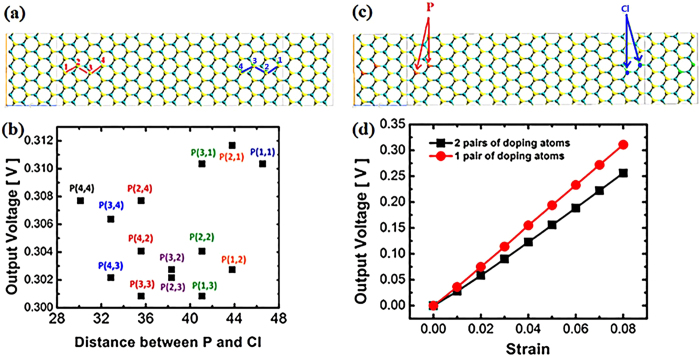
The investigation on doping effect. (**a**) The various combinational doping positions of P and Cl, denoted by P(m, n). As displayed, m, n are the atomic ordinal of P and Cl. (**b**) The output voltage (under 8% strain) as a function of doping position P(m, n). (**c**) Configuration of 2 pairs of doping atoms. (**d**) Comparison of output performance between 2 pairs of doping and 1 pair of doping structure.

Precisely control the dopant position and number is the main challenge for the application of low-dimensional nanomaterials. This inaccuracy in fabrication induces variations in mechanic and electronic properties of 2D materials and devices^48^,^49^. Therefore we investigate the variation of output voltage upon the various doping positions in MoS_2_ nanoribbon mechano-electric converter. As displayed by Fig. 7(a), the various combinational doping positions of P and Cl are denoted by P(m, n), where m, n are the atomic ordinal number of doping atoms P and Cl. P replaces S at left side while Cl is doping at right side. The variation of output voltage upon the doping combinations is exhibited in Fig. 7(b). The combination P(4,4) has the nearest distance between P and Cl while they reaches farthest away from each other in P(1,1). Generally the output voltage randomly fluctuates within small range from 0.301 V to 0.312 V. This limited variation modulated by various doping positions is favourable for future industrial applications. The doping concentration effect are also revealed in our study. 2 pairs of doping atoms are introduced in our device, as shown in Fig. 7(c). The dependence of output voltage on strain is demonstrated in Fig. 7(d). It increases linearly as larger strain. However, the output is lowered by higher doping concentration compared to that of the device based on one pair of doping atoms. The output is 0.028 V and 0.256 V for the device applied by 1% and 8% strain, respectively.

## TMDCs heterojunction based device

**Figure 8:**
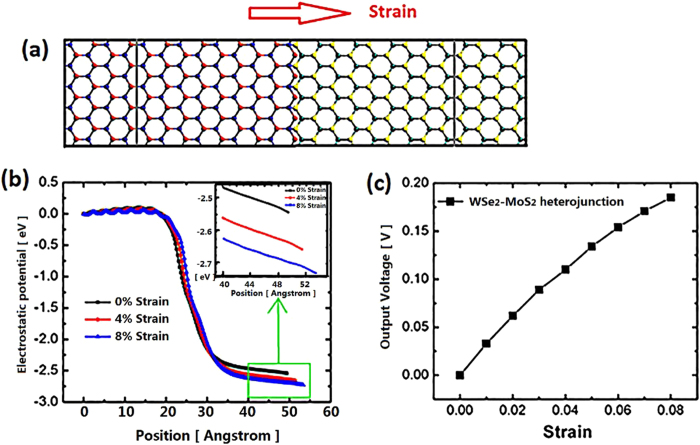
(**a**) Configuration of nano-power generator based on WSe_2_ and MoS_2_ heterojunction. The left part consists of WSe_2_ and the right part is MoS_2_, where Se atoms are blue, W atoms are red, S atoms are yellow and Mo atom are cyan. (**b**) 0%, 4% and 8% tensile strain modulated electrostatic potential along transport direction in the central region for device displayed in Fig. 5(a). Inset: the enlarged view of electrostatic potential within the region from 40 Å to 55 Å. (**c**) The evolution of the output voltage with strain.

We also investigate the mechano-electric converter based on TMDCs heterojunctions. Fig. 8(a). displayed the WSe_2_-MoS_2_ heterojunction based mechano-electric generator. The left part is WSe_2_ nanoribbon and the right counterpart is MoS_2_ nanoribbon. Fig. 8(b) reveals the electrostatic potential distribution along transport direction in the central region for the device under 0%, 4% and 8% tensile strain, respectively. The EPD mainly occurs at the narrow connection region between WSe_2_ and MoS_2_ nanoribbon. This radical change in electrostatic potential arises from the great difference between the work functions of WSe_2_ and MoS_2_ monolayer. As opposite to the regulatory change of EPD by strain in MoS_2_ PN junction, increasing strain causes larger EPD in heterojunctions. Fig. 8(c) reveals the output performance as a function of strain. The output voltage increases with larger strain and 0.185 V can be achieved by 8% strain. Our observation suggests that MoS_2_ PN junction based device has better output performance than TMDCs based heterojunctions.

**Figure 9:**
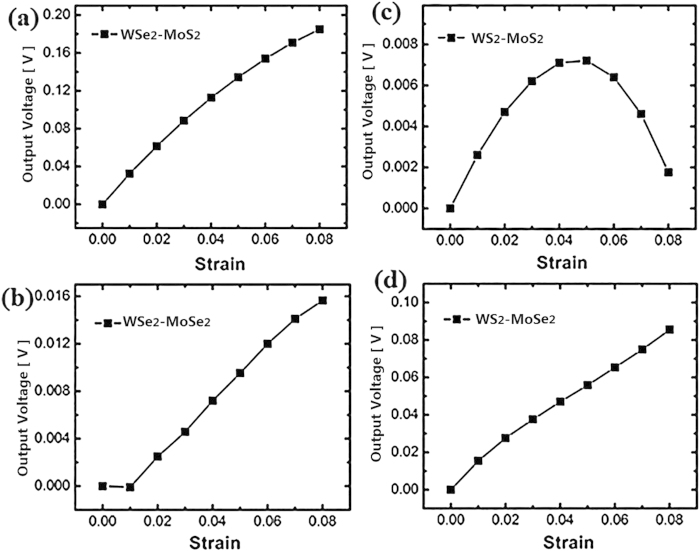
The output voltage as a function of strain for 4 heterojunction structures. (**a**) WSe_2_-MoS_2_ (**b**) WSe_2_-MoSe_2_ (**c**) WS_2_-MoS_2_ (**d**) WS_2_-MoSe_2_

Fig. 9 shows the output voltage as a function of strain for 4 different heterojunctions: a) WSe_2_-MoS_2_ (b) WSe_2_-MoSe_2_ (c) WS_2_-MoS_2_ (d) WS_2_-MoSe_2_. As shown in Fig. 9(c), WS_2_-MoS_2_ heterojunction reaches maximum output of 7.21 × 10^−3^ V under 5% strain, then reduces significantly by larger strain. For the other 3 structures, the output voltage is generally increasing by larger strain. Among these structures, WSe_2_-MoS_2_ heterojunction achieves the largest output of 0.185 V under 8% strain. However, this is still inferior to the performance of MoS_2_ PN junction, which possesses the output of 0.310 V under the equal strain. WSe_2_-MoS_2_ and WS_2_-MoSe_2_ can obtain the output voltage with a value of one order of magnitude larger than those of the other two structures, indicating that the enhanced output can be achieved by the TMDCs heterojunction based on different chalcogen materials. Table 1 summarizes the output voltage and EPD for 8 different TMDCs PN junctions and heterojunctions, all of which are in the same size of 5 × 9 (N_a_ = 5, N_b_ = 9). The heterojunction structures with higher EPD can achieve higher output voltage. Larger EPD is attributed to larger difference in work functions of distinct nanoribbons at opposite sides. This rule is also applied appropriately to the TMDCs PN junctions, among which the output voltage and EPD both reach largest value in WS_2_ PN junction.

## Summary

In summary, enlightened by the intrinsic piezoelectricity of TMDCs based two dimensional monolayer, we have designed and simulated a novel piezoelectric device realized by MoS_2_ monolayer based PN junction. Its electromechanical property was simulated by first-principle calculations. 0.31 V of output voltage can be achieved by 0.051 eV/ Å ^3^ of the laterally tensile stress, which leads to 8% strain in transport direction. We have also demonstrated the time domain-output voltage in the case of the applied stress in Sine waveform. The investigation on size-dependent performance demonstrates that by increasing width, length and layer number the output will finally converge to constant output. Our investigation on the doping effect shows that various doping positions affect slightly on the output voltage and the low concentration gives rise to higher output performance. The piezoelectric performance based on 4 different TMDCs-heterojunction were also simulated. We conclude that the structure with higher EPD can obtain higher output voltage. Our study suggests a novel TMDCs PN junction and heterojunction based mechano-electric generator with high output voltage. This may open up a suite of applications in 2D-TMDCs based piezoelectric transistor.

## Additional Information

**How to cite this article**: Yu, S. *et al.* Novel Two-Dimensional Mechano-Electric Generators and Sensors Based on Transition Metal Dichalcogenides. *Sci. Rep.* **5**, 12854; doi: 10.1038/srep12854 (2015).

## Figures and Tables

**Figure 1:**
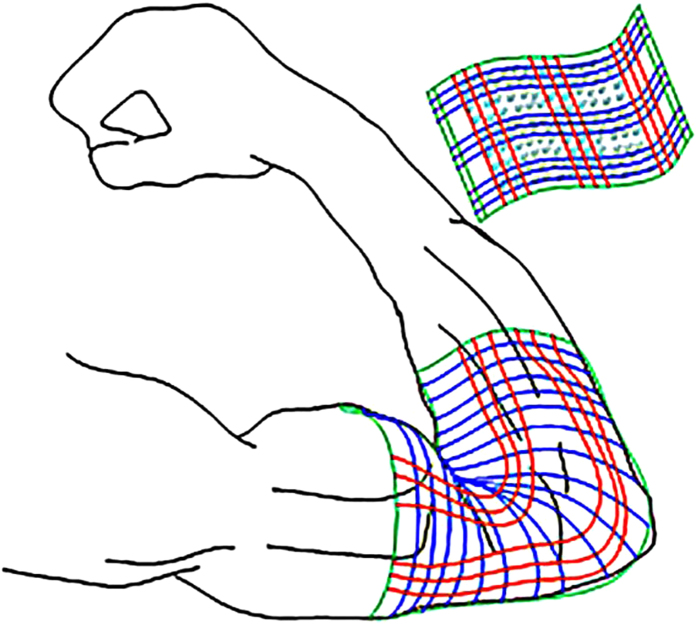
Carry-on electronics with mechano-electric generator based on 2D semiconductors.

**Table 1:** The comparison of output voltage and EPD for different structures.

**Strain** **=** **8%**	**Output [ V ]**	**EPD [ eV ]**
MoS_2_ PN junction	0.310	1.174
WSe_2_ PN junction	0.328	1.089
MoSe_2_ PN junction	0.189	0.992
WS_2_ PN junction	0.356	1.369
WSe_2_-MoS_2_ heterojunction	0.185	2.543
WS_2_-MoS_2_ heterojunction	0.00721	0.296
WS_2_-MoSe_2_ heterojunction	0.0855	2.377
WSe_2_-MoSe_2_ heterojunction	0.0157	0.215
